# Antidepressant-Like Effects of Acupuncture-Insights From DNA Methylation and Histone Modifications of Brain-Derived Neurotrophic Factor

**DOI:** 10.3389/fpsyt.2018.00102

**Published:** 2018-03-27

**Authors:** Huili Jiang, Xuhui Zhang, Jun Lu, Hong Meng, Yang Sun, Xinjing Yang, Bingcong Zhao, Tuya Bao

**Affiliations:** ^1^School of Acupuncture-Moxibustion and Tuina, Beijing University of Chinese Medicine, Beijing, China; ^2^Research Center of Mental and Neurological Disorders, School of Acupuncture-Moxibustion and Tuina, Beijing University of Chinese Medicine, Beijing, China; ^3^School of Science, Beijing Technology and Business University, Beijing, China

**Keywords:** acupuncture, brain-derived neurotrophic factor, depression, DNA methylation, histone modifications

## Abstract

Sensitive and stable biomarkers that facilitate depression detection and monitor the antidepressant efficiency are currently unavailable. Thus, the objective is to investigate the potential of DNA methylation and histone modifications of brain-derived neurotrophic factor (BDNF) in monitoring severity and antidepressive effects of acupuncture. The depression rat model was imitated by social isolation and chronic unpredicted mild stress (CUMS). The expression of serum BDNF was detected by enzyme-linked immunosorbent assay (ELISA), the hippocampal BDNF, acetylation levels in histone H3 lysine 9 (acH3K9), and HDAC2 by Western blot, the hippocampal mRNA of BDNF by RT-polymerase chain reaction (PCR). The DNA methylation patterns of the promoter I of BDNF was detected by MS-PCR. We investigated that the expression of BDNF in serum and hippocampus were significantly downregulated compared with controls. The same trend was found in mRNA of BDNF. Notably, acupuncture reversed the downregulation of BDNF in serum and hippocampus and mRNA of BDNF compared with model group. Acupuncture reversed the CUMS-induced downregulation of hippocampal acH3K9. On the contrary, the CUMS-induced upregulation of hippocampal HDAC2 in model group was significantly reversed by acupuncture. Collectively, the antidepressant effect of acupuncture might be mediated by regulating the DNA methylation and histone modifications of BDNF, which may represent novel biomaker for detection of depression and monitoring severity and antidepressive effects.

## Introduction

Depression, one of the most prevalent psychiatric disorders, is considered to be one of the leading causes of disability (1, 2). Stress could trigger the behavioral and psychological modifications and induce the alterations of homeostasis and internal balance of the organism. Increasing evidence suggests that chronic stress is one of the leading contributing factors in the development, maintenance, or exacerbation of depression (3–6). Data from clinical investigations and laboratory animals suggest that the systemic perturbations of homeostasis could induce the dysfunction of abnormalities in regional brain activity (7, 8), impaired neurogenesis, and changes in synaptic function (9–12).

Currently, the precise molecular mechanisms responsible for the pathogenesis of depression remain to be not further clarified. However, extensive evidence has associated a variety of intracellular pathways and signal transduction cascades with the pathophysiology and treatment of depression. There is also preliminary evidence that depression is the result of a combined effect of stressful factors and genetic susceptibility (13–16). As already mentioned, chronic stress to genetically vulnerable individuals might induce the dysfunctions of neurogenesis and synaptic function, or a significant reduction of brain-derived neurotrophic factor (BDNF) expression (17, 18). BDNF, a member of the neurotrophin family, has been considered to contribute to the nervous system development and function. And it has been identified that BDNF is involved in the pathogenesis of a wide range of psychiatric disorders, including depression (19, 20), schizophrenia, and anxiety disorder. Recently, it has been shown that BDNF is widely distributed throughout the mammalian brain, which includes the hippocampus, cerebral cortex, basal forebrain, limbic structures, and cerebellum, etc. Numerous studies have associated BDNF with reward-related processes, learning and memory, cognitive function, and circuit formation. Notably, data from clinical investigations and laboratory animals have provided compelling evidence that BDNF is involved in the pathogenesis of depression (19–21).

Recent evidence has identified that epigenetic mechanisms, including DNA methylation, histone modifications, and non-coding RNAs, are considered to be able to reliably differentiate the stable and heritable characteristics of depression (22–24). Epigenetic mechanisms play an important role in the BDNF expression regulation. The preliminary investigation of epigenetic modification has been carried out focusing on DNA methylation. It has been shown that DNA methylation is the first described and the most investigated epigenetic modification (22). Of note, the DNA methylation level of BDNF is identified to be significantly important in monitoring the severity of symptoms as well as antidepressant effect (25–27). Previous studies have shed light on the potential possibility of the DNA methylation expression level as a biomarker of major depressive disorder (MDD) (28). It has been widely accepted that the DNA methylation level and the DNA methylation patterns of the promoter level could affect gene expression level. Data from subsequent investigation has verified a significantly upregulated methylation level of the analyzed region inside of BDNF promoter I as well as a significant decrease of BDNF expression in MDD patients. Other studies have investigated the expression level of DNA methylation of BDNF in treatment-resistant major depressive patients, and the different expression in the methylation level of BDNF promoter I has been investigated (29). During our previous studies, we have been focusing on investigating the clinical effects and mechanisms of acupuncture on depression. Our preliminary findings have indicated that acupuncture could reverse the stress-induced decrease of BDNF expression and exert antidepressant effect, which has also been supported by the data from clinical investigations. However, the epigenetic mechanisms underlying the antidepressant effect of acupuncture by regulating the methylation level of the analyzed region inside of BDNF promoter I and BDNF expression have not been investigated in depth.

Accordingly, here we established the depression rat model imitated by social isolation and chronic unpredicted mild stress (CUMS) procedures and investigated the epigenetic mechanisms underlying the antidepressant effect of acupuncture by regulating the methylation level of the analyzed region inside of BDNF promoter I and BDNF expression. The expression of serum BDNF was detected by enzyme-linked immunosorbent assay (ELISA), the hippocampal BDNF, acH3K9 and HDAC2 by Western blot (WB), the hippocampal mRNA of BDNF by RT-polymerase chain reaction (PCR). The DNA methylation patterns of the promoter I of BDNF was detected by MS-PCR. We aimed to elucidate the epigenetic mechanisms underlying the antidepressant effect of acupuncture and investigated the potential of DNA methylation and histone modifications of BDNF in monitoring severity of symptoms and antidepressant effect of acupuncture, which might shed new light on conceptual frameworks of prospects for new therapies in the treatment of depression.

## Materials and Methods

### Animals

Five-week-old male Sprague–Dawley rats, weighting (220 ± 20) g, were obtained from the Weitong Lihua Experimental Animal Center (Beijing, China). All rats were housed in a controlled environment of 23–26°C, 50 ± 10% humidity, and in quiet states. The rats had access *ad libitum* to standard rat chow and tap water. The rats exposed to CUMS were housed separately in different cages for social isolation and five animals per cage were housed for rats in the control group. All experimental procedures were in full observance of the International Association for the Use of Animals in Research, and approved by the Institute of Animal Care Committee of the Beijing University of Chinese Medicine (Permit No. Kj-dw-32-20151012).

### Experimental Grouping

**Figure 1:**
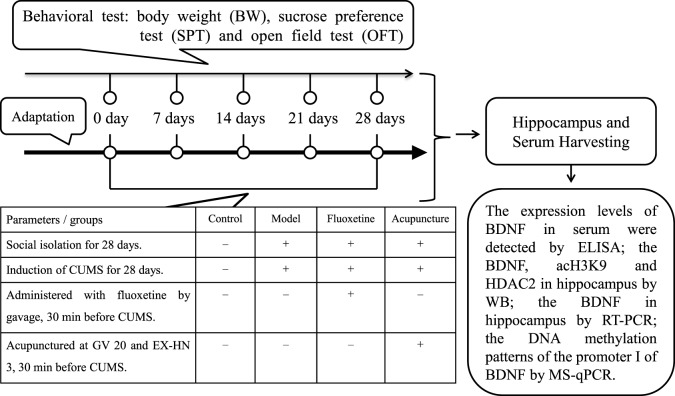
Experimental procedures. CUMS, chronic unpredicted mild stress; BDNF, brain-derived neurotrophic factor; acH3K9, acetylation levels in histone H3 lysine 9; HDAC2, histone deacetylase 2; RT-polymerase chain reaction (PCR), reverse transcription-polymerase chain reaction; MS-qPCR, methylation-specific quantitative polymerase chain reaction; BW, body weight; SPT, sucrose preference test; OFT, open field test.

The body weight (BW), sucrose preference test (SPT), and open field test (OFT) were observed to guarantee the consistency of baseline characteristics before the experimental guidelines was implemented. Nine rats were excluded due to the inconsistent baseline characteristics. Thus, a total of 40 rats under the circumstance of similar baseline characteristics of BW, SPT, and OFT were enrolled in the final analysis. Rats were randomly assigned into control group, model group, fluoxetine group (FLX), and acupuncture group (Acu) (see Figure 1), with 10 rats in each group. Rats in model, fluoxetine, and acupuncture groups were subjected to social isolation and CUMS for 28 days excluding rats in control group. 30 min before CUMS procedure, rats in FLX group were administered with fluoxetine (1.8 mg/kg with the concentration of 0.18 mg/ml distilled water) by gavage (1 ml/100 g) once daily, for 28 days. And rats in acupuncture group were acupunctured at *Baihui* (GV 20) and *Yintang* (GV 29) once daily, for 28 days.

### Reagents and Antibodies

In this study, sucrose (Amresco; 20140319) was employed in the sucrose consumption test, and fluoxetine (Eli Lilly and Company, Suzhou 215021, China; 4126A) was used as the standard control for acupuncture. ELISA Kit of BDNF (BlueGene Biotech CO., LTD., China; E02B0029) was used to detect the expression of BDNF in serum. The antibodies and some reagents used for WB analysis included rabbit polyclonal anti-BDNF (LifeSpan BioSciences; LS-C343943), rabbit polyclonal anti-acH3K9 (Cell Signaling Technology; 9649S), mouse anti-HDAC2 (Cell Signaling Technology; 5113S), β-actin (Zhongshanjinqiao Biotechnology Co., Ltd., Beijing, China; TA-09), goat anti-rabbit IgG (Jackson; 111-035-003), rabbit anti-H3 (Cell Signaling Technology, 4499S), ECL (Millipore; WBKLS0500), and bicinchoninic acid (BCA) protein assay kit (Cwbiotech; 02912E). Furthermore, the reagents used for RT-PCR and methylation-specific quantitative real-time PCR analysis included ultrapure RNA kit (Cwbiotech), HiFi-MMLV Reverse Transcriptase (Cwbiotech), SYBR Green PCR Mixture (Cwbiotech), and Dnase I (Cwbiotech), etc.

### Induction of CUMS

The rat model of depression was simulated by CUMS as described previously (10, 30). Meanwhile, some available adjustments were made to fortify the unpredictability. The rats were exposed to CUMS for 28 days (10, 31), including housing in a wet cage for 24 h (containing 100 g of sawdust in 200 ml water), restricted access to food deprivation for 24 h, continuous water deprivation for 24 h, continuous overnight illumination for 12 h, restricted access to chronic restraint stress for 2 h (restraining in a cylinder-shaped wire net, 20-cm length and 5-cm diameter), ice water swimming at 4°C for 5 min, clip tail for 3 min (1 cm apart from the tail). Rats exposed to CUMS were subjected to one of these seven stimuli at random per day (Table 1). Importantly, the same stressor was not used on consecutive days to avoid rat’s prediction. Each stressor was employed in the CUMS procedure three times randomly. Rats in the control group were normally fed for 28 days with food and water *ad libitum* without any stimulus.

**Table 1:** The chronic unpredicted mild stress procedure of applied stressors during 1 week.

Days	Duration	Stressor
Day 1	5 min	Ice water swimming at 4°C
Day 2	2 h	Chronic restraint stress
Day 3	3 min	Clip tail
Day 4	24 h	Housing in a wet cage
Day 5	12 h	Continuous overnight illumination
Day 6	24 h	Water deprivation
Day 7	24 h	Food deprivation

### Acupuncture Stimulation

Thirty minutes before CUMS procedure, rats in acupuncture group were subjected to acupuncture stimulation, 20 min per session, one session daily for 28 days. Following disinfection of the acupoint sites with 75% alcohol, the acupuncture needles (0.3 mm in diameter and 25 mm long; Suzhou Acupuncture & Moxibustion Appliance Co., Ltd., Jiangsu, China) were inserted transversely (keeping the angle between the needle and the skin surface at 15 angle) into *Baihui* (GV 20) and *Yintang* (GV 29) to a depth of 5 mm as described by our previous study (10). Acupoints coordinates [GV 20, located at the bregma, or on the junction of coronal suture and sagittal suture; GV 29, midway between the medial ends of the two eyebrows] were under the guidance as described previously (32). When acupuncture stimulation was carried out, rats were placed in separate room under the conditions of free activities. Additional stress was strictly avoided during each procedure.

### Behavioral Observation

All observations were conducted under relatively quiet and dark circumstances. All behavioral tests were performed by the same examiner who was blinded to the group allocations. BW, SPT, and OFT were investigated at least 12 h after the stress stimulation at the end of experimental period. Mood states, quality of feces, and appetite of rats were also observed and recorded.

### Body Weight

The alterations in BW gain in comparison with the baseline were detected to evaluate the states of food preference and nutrition status. BW was detected at 0 day pre-intervention and at 7, 14, 21, and 28 days post-intervention for each rat throughout the experimental procedures.

### Sucrose Preference Test

Sucrose preference was evaluated in all rats by calculating the reduction in the consumption of sucrose solution or preference as an indicator of anhedonia (10, 30). All rats were trained to adapt to 1% sucrose solution during the adaptation procedures. Specifically, following a 16 h period of food and water deprivation, all rats were presented simultaneously with two pre-weighed bottles, one bottle containing sucrose (1% w/v) and the other containing water, for a period of 60 min. Of note, the original order of the two bottles was reversed randomly from trial-to-trial, during the training procedures. After the training period, all rats were deprived of food and water for 23 h. Then they were all housed in individual cages and had free access to two pre-weighed bottles containing 150 ml sucrose solution (1% w/v) and 150 ml pure water for 1 h. At the end of the test, the bottles of 1% sucrose solution and pure water were re-weighted and recorded to calculate amount consumed from each solution. Sucrose preference was calculated from the following formula (33): Sucrose preference = {Sucrose intake (g)/[Sucrose intake (g) + Water intake (g)]} × 100%. SPT was measured at 0 day pre-intervention and at 7, 14, 21, and 28 days post-intervention in each rat throughout the experimental procedures. Anhedonia was expressed by reduced sucrose preference.

### Open Field Test

General locomotor activity of each rat was detected through OFT as described by previous studies (10, 34). The open field apparatus consisted an 80 × 80 × 40 cm square arena with black walls and black base, of which the base was divided into 16 × 16 cm equal squares with legible white strips. Each rat was gently placed in the center of the open field floor and then allowed to enjoy independent movement and explore freely for 5 min. Crawling square numbers (numbers of crossing the horizontal sectors including three paws in the same square) and standing times (numbers of erection including rearing) were monitored and recorded as an index of general locomotion activity and exploratory behavior. After each trial, 75% ethyl alcohol was used to refresh the open field apparatus to avoid the interference of odor signals left behind by the other rats. OFT was performed at 0 day pre-intervention and at 7, 14, 21, and 28 days post-intervention in all the rats manually during each session. Locomotor activity scores, the comprehensive analysis of the horizontal and vertical motion scores, were used to evaluate the ability of the rats to adapt to a new environment and the general locomotor activities.

### Samples Collection

After 28 days of experimental intervention, rats in the four groups were sacrificed on day 29. The animals were anesthetized with an injection of 10% chloral hydrate (0.35 mL/100 g, i.p.) followed by blood samples collection. Then the blood samples were left to coagulate and centrifuged at 1,000 × *g* for 10 min to separate serum. Serum samples were then stored at −20°C for the next experimental process. Rats were then sacrificed by decapitation and the hippocampus was dissected under strict cooling conditions. The samples were then snap frozen in liquid nitrogen and placed in a freezer at −80°C for the next experiments.

### Biochemical Assays

#### Determination of BDNF in Serum

The expression levels of BDNF in serum were quantified using the corresponding ELISA. The procedure was performed according to instructions of the ELISA kit provided by the manufacturer. Levels were expressed as pg/mL.

#### WB Analysis for BDNF, acH3K9, and HDAC2 in Hippocampus

The procedures of western blot (WB) were as follows. The samples were homogenized with RIPA lysis buffer, containing 50 mM Tris (pH 7.4), 150 mM NaCl, 1% NP-40 and 0.5% Na-deoxycholate, and protease inhibitor cocktail for protein extraction. And then the supernatant was collected following centrifugation at 13,000 rpm at 4°C for 20 min. The total protein content was determined by using BCA assay. Following the quantitative determination of total protein content, the proteins of each sample were denatured at 100°C for 5 min. Subsequently, protein samples were fractionated through 10% SDS-polyacrylamide gel electrophoresis. The proteins of samples were electrotransferred onto polyvinylidene difluoride membranes with voltage at 80 V for 60 min. The membranes were blocked with 5% bull serum albumin-TBST for 1 h at room temperature. Protein expression was subsequently detected by incubation with rabbit polyclonal primary antibodies against BDNF (1:1,000) and β-actin (1:1,000); acH3K9 (1:1,000) and histone H3 (1:1,000); HDAC2 (1:1,000) and histone H3 (1:1,000), at 4°C overnight. Following incubation with the primary antibody, the membranes were incubated with Goat anti-rabbit HRP-conjugated IgG (1:10,000) at room temperature for 40 min. The bound antibodies were visualized using an enhanced chemiluminescence Reagent by ECL kit (RPN2232; GE Healthcare Life Sciences, UK) and quantified densitometrically using Gel-image analyzing system (Gene gnome, Syngene, USA). The experiments were performed in triplicate with triplicate samples. In the WB analysis, histone H3 as loading control was used to normalize the levels of nuclear protein of HDAC2 and acH3K9 detected; and β-actin as loading control was used to normalize the levels of protein of BDNF detected. The mean optical density value of each protein band relative to that of the β-actin band from the same sample was calculated.

#### Real-Time Reverse Transcription-Polymerase Chain Reaction

The total RNA of the hippocampus was extracted using Trizol Reagent containing guanidium thiocyanate according to the manufacturer’s instruction. RNA was quantified by spectrophotometric analysis (OD 260/280). The reverse transcription reagent kit was used to obtain first strand cDNA. For real-time PCR analysis, the cDNA samples were run in triplicate and β-actin was used as a reference gene. Each PCR amplification included non-template controls and all reagents except for the cDNA. Real-time PCR reactions were performed with Taq polymerase using SYBR Green real-time PCR method. The operating steps of PCR amplifications were conducted according to instructions of the manufacturer. For a reference gene, β-actin was used. The reactions of BDNF (total volume, 20 µl) were conducted with typical thermal profile of pre-incubation at 95°C for 10 min, followed by 45 cycles of 95°C for 10 s and 59°C for 60 s. Melting curve analysis was performed at the end of each run for each primer pair, allowing us to control amplification specificity. After PCR amplification, the comparative Ct method, also called ΔCt, is calculated by formula as follows (35): ΔCt = avgCt _GOI_−avg Ct _ref_. GOI indicated gene of interest; ref indicated the reference gene. The ΔCt was calculated by subtracting the β-actin Ct from each sample Ct. The products of PCR were detected by agarose gel electrophoresis, which was observed under ultraviolet light and photographed. Primers were designed using the Primer-Blast program obtained from NCBI. The primer sets used were as the following: BDNF: forward: F5′-TAGCAAAAAGAGAATTGGCTG-3′, reverse: R5′-TTTCAGGTCATGGATATGTCC-3′; β-actin: forward: F5′-TCATGAAGTGTGACGTTGACATCCGTAAAG-3′, reverse: R5′-CGTAGAAGCATTTGCGGTGCACGATGGAGG-3′, with the predicted sizes 255 and 283 bp, respectively.

#### Methylation-Specific Real-Time PCR

**Figure 2:**
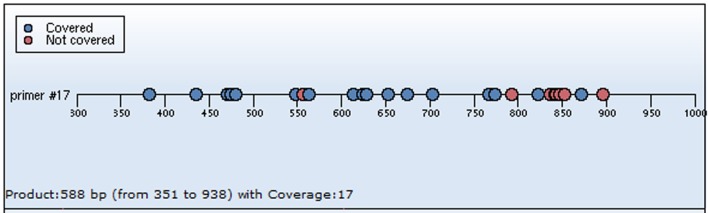
Primers were designed based on the sequenom ^®^ EpiDesigner software system. The CpG sites at CpG Island one were involved in the primers design, performed with a program of BDNF #17 was analyzed by the procedures. Each dot indicated one CpG site: the blue dots indicated covered CpG sites, and the red dot covered CpG sites. In this study, the 12 CpG sites situated in the BDNF promoter I are as follows: CpG2, CpG4, CpG5, CpG6, CpG10, CpG11, CpG12, CpG13, CpG14, CpG15, CpG16, CpG18, and CpG24.

The procedures of the methylation-specific real-time PCR are as follows (36–38). Following the extraction of DNA from 100 mg hippocampus of all samples, DNA concentration and purity was detected by spectrophotometry. Then the DNA deamination was performed according to the manufacturer’s instructions for the next procedure. The modified DNA was used as a template for methylation-specific real-time PCR. The operating steps of quantitative real-time PCR were conducted according to instructions of the manufacturer. Primers were designed based on the EpiDesigner software system (see Figure 2). The primers were used to detect methylated gene of the BDNF promoter I at relative CpG sites. β-actin gene was used as a reference control. The relative BDNF promoter I methylation level was expressed as the ratio of methylated gene of the BDNF promoter I DNA to β-actin DNA. Details were as follows. All reactions were run in a volume of 5 µl on a microtiter plate. The reaction mixture included 1 µl of deaminated DNA, 0.04 µl dNTP mix, and 0.1 µl two sets of primers at a final concentration of 10 pmol/μL each. All reactions were performed in triplicate. As far as the qPCR settings were concerned, initial denaturation was performed at 94°C for 4 min, followed by amplification at 94°C for 20 s, 56°C for 30 s, 72°C for 60 s for 45 cycles. Then the melting curve analysis was carried out as the standard procedure indicated, at 94°C for 5 s, followed by cooling to 52°C for 5 s for 40 cycles, followed by heating to 80°C for 5 s for 5 cycles, finally followed by cooling to 72°C for 3 min. Intra-assay variation and inter-assay variation were evaluated for quality control. Standard curves were generated based on the results and were applied for quantitative data analysis. The primer sets used were as follows: BNDF #17: forward: aggaagagagGGGGTTAGGGTAGTTTTTTTGAGT; reverse: cagtaatacgactcactatagggagaaggctCCAAAACCCACCTTCTAAAAC, with the predicted size 555 bp and coverage 17.

### Data Analysis

All statistical tests were conducted using SPSS 22.0 software package (IBM, Armonk, New York, NY, USA). Data were presented as mean with standard deviation (mean ± SD). Within each group, the data of BW gain, sucrose consumption, and general locomotor activity at different time points were analyzed by two-way analysis of variance (ANOVA) with Tukey’s *post hoc* test. Additionally, one-way ANOVA were used. Differences between individual means were tested for significance using Fisher’s least significant difference procedure. Linear regression analysis and bivariate correlation analysis was performed to elucidate the correlation test among the indexes. Significant threshold was set at *P* < 0.05.

## Results

### Behavioral Observations

#### Changes in BW

**Figure 3:**
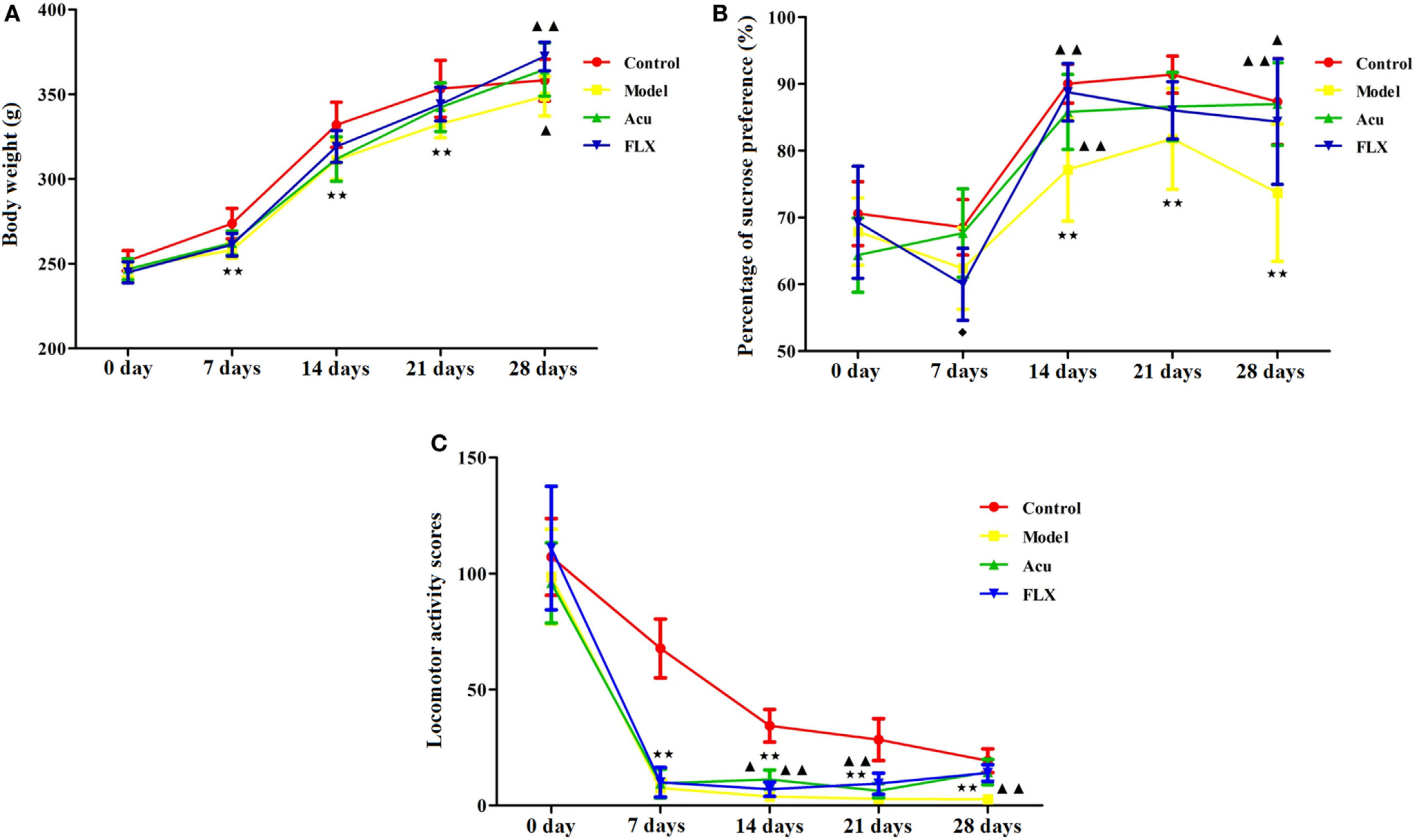
Differences showing the effects of stress/antidepressant treatments on depressive-like behaviors in depression rats induced by chronic unpredicted mild stress. Acu, acupuncture group; FLX, fluoxetine group. Differences were assessed with two-way analysis of variance (ANOVA) with Tukey’s *post hoc* test. Results were presented as mean ± SD. **(A)** Results of two-way ANOVA with time points and groups of changes in BW were as follows: time points, *F* = 4.142, *P* = 0.000; group, *F* = 8.794, *P* = 0.000; interaction, *F* = 1,220.311, *P* = 0.000. ^★^^★^*P* < 0.01 compared with the control group; ^▲^*P* < 0.05 compared with the model group; ^▲▲^*P* < 0.01 compared with the model group. **(B)** Results of two-way ANOVA with time points and groups on changes in sucrose consumption were as follows: time points, *F* = 107.438, *P* = 0.000; group, *F* = 20.266, *P* = 0.000; interaction, *F* = 2.788, *P* = 0.000. ^★^^★^*P* < 0.01 compared with the control group; ^▲^*P* < 0.05 compared with the model group; ^▲▲^*P* < 0.01 compared with the model group; ^◆^*P* < 0.05 compared with the acupuncture group. **(C)** Results of two-way ANOVA with time points and groups on changes in locomotor activity scores were as follows: time points, *F* = 569.024, *P* = 0.000; group, *F* = 68.783, *P* = 0.000; interaction, *F* = 11.279, *P* = 0.000. ^★^^★^*P* < 0.01 compared with the control group; ^▲^*P* < 0.05, compared with the model group; ^▲▲^*P* < 0.01 compared with the model group; ^◆^*P* < 0.01 compared with the acupuncture group.

Before the experimental procedures, there was no significant difference among groups. However, significant differences in the changes of BW were found among groups after the CUMS procedures and intervention. Rats in the model group were screened to eat less with poor appetite, and exhibit low spirits, fur shedding, and reduced luster. The weight of the rats in the model group was significantly lower than that of the rats in the control group at 7, 14, and 21 days during the CUMS procedure (*P* < 0.01; *P* < 0.01; *P* < 0.01). In comparison with the model group, rats in fluoxetine and acupuncture groups gained more weight after the intervention (*P* < 0.01; *P* < 0.05). There were no significant differences in responses to BW gain between fluoxetine and acupuncture groups (Figure 3A).

#### Sucrose Preference Test

Significant differences were found in sucrose consumption-related indicators between the control and model groups, especially at 14, 21, and 28 days of the CUMS procedure (*P* < 0.01; *P* < 0.01; *P* < 0.01). Rats in the model group exhibited the reduced sensitivity to reward stimulation and pleasure. However, both fluoxetine and acupuncture significantly reversed the decreased sucrose consumption when compared with that in the model group, at 14 and 28 days of the experimental procedure with statistical significance (*P* < 0.01, *P* < 0.05; *P* < 0.01, *P* < 0.01). Significant difference was also found between fluoxetine and acupuncture groups at 7 days of the experimental procedure (*P* < 0.05) (Figure 3B).

#### Open Field Test

Results of the OFT showed that rats in the model and control groups exhibited significant differences in the locomotor activity scores (comprehensive analysis of the horizontal and vertical motion scores), especially at 7, 14, 21, and 28 days of the CUMS procedure (*P* < 0.01; *P* < 0.01; *P* < 0.01; *P* < 0.01). And the ability of the rats in the model group to adapt to a new environment was significantly decreased. Of note, the locomotor activity scores of the rats in the fluoxetine and acupuncture groups were notably elevated following the intervention of fluoxetine and acupuncture at 14, 21, and 28 days of the experimental procedure, with statistical significance (*P* < 0.05, *P* < 0.01, *P* < 0.01; *P* < 0.01, *P* < 0.01, *P* < 0.01). Significant difference was also found between fluoxetine and acupuncture groups at 14 days of the experimental procedure (*P* < 0.01) (Figure 3C).

#### Expression Level of BDNF in Serum

**Figure 4:**
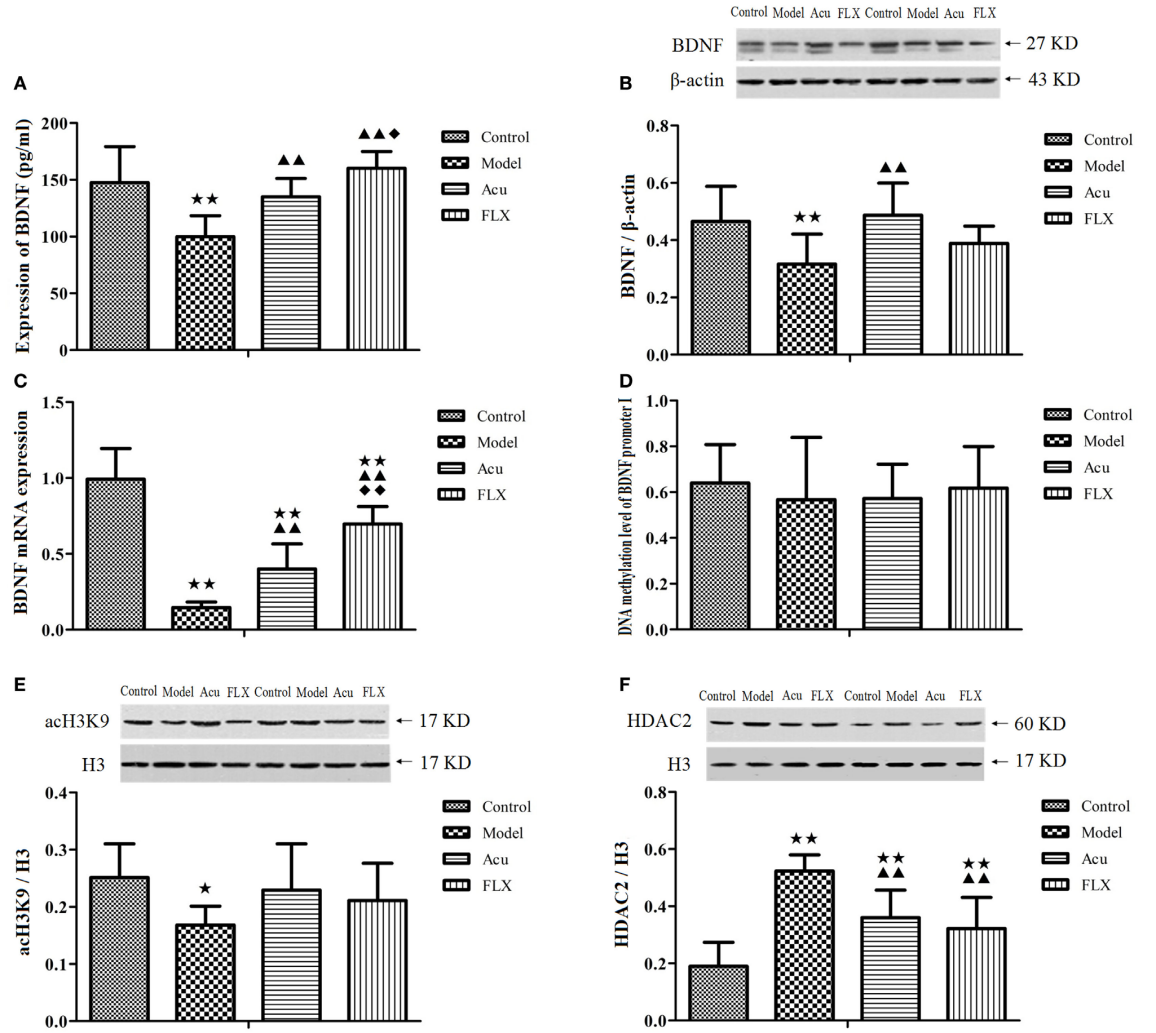

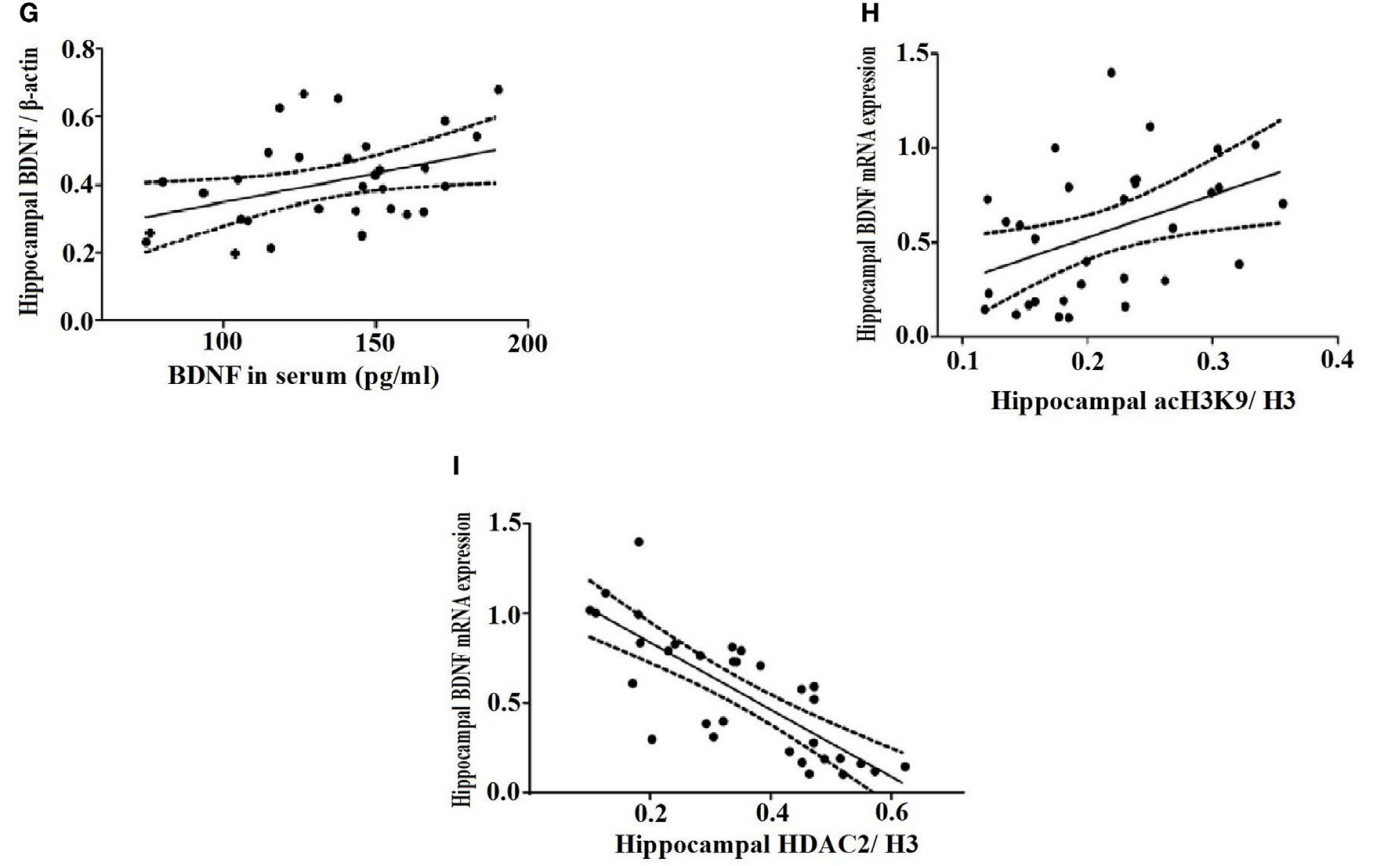
Differences showing the effects of stress/antidepressant treatments on the expression of DNA methylation and histone modifications levels of BDNF in depression rats induced by chronic unpredicted mild stress. Acu, acupuncture group; FLX, fluoxetine group; BDNF, brain-derived neurotrophic factor; RT-PCR, real-time reverse transcription-polymerase chain reaction; MS-PCR, methylation-specific polymerase chain reaction; acH3K9, acetylation levels of Histone 3 at Lysine 9; HDAC2, histone deacetylase 2. Differences were assessed with one-way analysis of variance with Fisher’s least significant difference (LSD) procedure. Results were presented as the mean ± SD **(A)** Enzyme-linked immunosorbent assay analysis of BDNF expression in serum of each group (*n* = 8). ^★^^★^*P* < 0.01 compared with the control group; ^▲▲^*P* < 0.01 compared with the model group; ^◆^*P* < 0.05 compared with the acupuncture group. **(B)** Western blot (WB) analysis of BDNF expression in hippocampus of each group (*n* = 8). ^★^^★^*P* < 0.01 compared with the control group; ^▲▲^*P* < 0.01 compared with the model group. **(C)** Expression of mRNA level of BDNF in the hippocampus of each group (*n* = 8). ^★^^★^*P* < 0.01 compared with the control group; ^▲▲^*P* < 0.01 compared with the model group; ^◆◆^*P* < 0.01 compared with the acupuncture group. **(D)** The DNA methylation level of hippocampal BDNF promoter I at relative CpG sites among each group (*n* = 6), as detected by MS-PCR. The 12 CpG sites situated in the BDNF promoter I are as follows: CpG2, CpG4, CpG 5, CpG6, CpG10, CpG11, CpG12, CpG13, CpG14, CpG15, CpG16, CpG18, and CpG24. **(E)** The expression level of acH3K9 in hippocampus among each group (*n* = 8), as detected by WB. ^★^*P* < 0.05 compared with the control group. **(F)** The expression level of HDAC2 in hippocampus among each group (*n* = 8), as detected by WB. ^★^^★^*P* < 0.01 compared with the control group; ^▲▲^*P* < 0.01 compared with the model group. **(G)** The correlation analysis between the expression level of BDNF in serum and hippocampus. Linear regression analysis: *r* = 0.386, R^2^ = 0.149; *F* = 5.250, *P* = 0.029; *t* = 2.291, *P* = 0.029, which indicated a positive correlation between the expression level of BDNF in serum and hippocampus. **(H)** The correlation analysis between the expression level of hippocampal BDNF mRNA and acH3K9. Linear regression analysis: *r* = 0.431, R^2^ = 0.185; *F* = 6.831, *P* = 0.014; *t* = 2.614, *P* = 0.014, which indicated a positive correlation between the expression level of hippocampal BDNF mRNA and acH3K9. **(I)** The correlation analysis between the expression level of hippocampal BDNF mRNA and HDAC2. Linear regression analysis: *r* = 0.787, R^2^ = 0.619; *F* = 48.783, *P* = 0.000; *t* = −6.985, *P* = 0.000, which indicated a significantly negative correlation between the expression level of hippocampal BDNF mRNA and HDAC2.

Compared with the control group, the expression of BDNF in serum in the model group was significantly downregulated (*P* < 0.01). Following the intervention of acupuncture and fluoxetine, the expression of BDNF in serum in acupuncture and FLXs were significantly upregulated compared with that in the model group (*P* < 0.01; *P* < 0.01) (Figure 4A). Both acupuncture and fluoxetine could reverse the downregulation of BDNF in serum induced by CUMS. Significance was also found between acupuncture and FLXs (*P* < 0.05) (Figure 4A).

#### Expression Level of BDNF in the Hippocampus

The result showed that, compared with the control group, the expression of hippocampal BDNF in the model group was significantly downregulated (*P* < 0.01). Of note, acupuncture reversed the downregulation of hippocampal BDNF when compared with that in the model group with statistical significance (*P* < 0.01). There were no significant differences in the expression of hippocampal BDNF between fluoxetine and model group (*P* > 0.05) (Figure 4B).

#### Expression of mRNA Level of BDNF in the Hippocampus

The results indicated that the relative mRNA expression level of BDNF in the hippocampus of the model group was significantly lower than that in the control group (*P* < 0.01). In comparison with the model group, the lower expression of mRNA levels of BDNF was significantly reversed in the acupuncture and FLXs following the intervention of acupuncture and fluoxetine (*P* < 0.01; *P* < 0.01). Significance was also found between acupuncture and FLXs (*P* < 0.01). Importantly, the relative mRNA expression level of BDNF in the hippocampus of the acupuncture group was evidently higher than that in the FLX (Figure 4C).

#### The DNA Methylation Level of Hippocampal BDNF Promoter I Corresponding to 12 CpG Sites

In this study, the DNA methylation level of hippocampal BDNF promoter I was assessed at 12 CpG sites, including CpG2, CpG4, CpG 5, CpG6, CpG10, CpG11, CpG12, CpG13, CpG14, CpG15, CpG16, CpG18, and CpG24, as detected by MS-PCR. In comparison with the DNA methylation level of BDNF promoter I at relative CpG sites, no significant differences have been found among control, model, acupuncture, and FLXs recently (*P* > 0.05). The precise mechanisms that the DNA methylation level of BDNF promoter I are involved in the pathogenesis of depression remains to be further clarified (Figure 4D).

#### Expression Level of acH3K9 and HDAC2 in Hippocampus

In the WB analysis, histone H3 as loading control was used to normalize the levels of nuclear protein detected. The expression level of hippocampal acH3K9 in the model group was significantly downregulated following CUMS procedures in comparison with that in the control group (*P* < 0.05). However, the expression level of hippocampal HDAC2 in the model group was increased in the model group when compared with that in the control group, with statistical significance (*P* < 0.01). There were no significant differences in the expression level of hippocampal acH3K9 among the model, acupuncture and FLXs (*P* > 0.05). In comparison with the model group, the upregulated expression levels of hippocampal HDAC2 were significantly reversed in the acupuncture and FLXs following the intervention of acupuncture and fluoxetine (*P* < 0.01; *P* < 0.01) (Figures 4E,F).

## Discussion

In this study, we identified some compelling findings on the antidepressant effect of acupuncture from the perspective of the molecular level. The rat model of depression in our study was induced by CUMS procedures, which has been evidenced to be parallel to the symptoms of depression and accurately recapitulate the human condition (10, 34, 39, 40). This study aimed to elucidate the epigenetic mechanisms underlying the antidepressant effect of acupuncture and investigated the potential of DNA methylation and histone modifications of BDNF in monitoring severity of symptoms and antidepressant effect of acupuncture, which might shed new light on conceptual frameworks of prospects for new therapies in the treatment of depression.

### The Antidepressant Effect of Acupuncture

The alterations in BW gain in comparison with the baseline were detected to evaluate the states of food preference and nutrition status. BW was detected at 0 day pre-intervention and at 7, 14, 21, and 28 days post-intervention for each rat throughout the experimental procedures. Before the experimental procedures, there was no significant difference among groups. However, significant differences in the changes of BW were found among groups after the CUMS procedures and intervention. Rats in the model group were screened to eat less with poor appetite, and exhibit low spirits, fur shedding, and reduced luster. The weight of the rats in the model group was significantly lower than that of the rats in the control group. In comparison with the model group, rats in fluoxetine and acupuncture gained more weight after the intervention. Although the statistical difference was not significantly remarkable, significance was indeed found in the stress-induced less BW gain in this study. Importantly, we found acupuncture reversed it. The result of the present study is consistent with the current study illustrated that CUMS procedures could induce depressive-like behaviors, well imitating the symptom of depression. BW or body mass index (BMI) has been considered of the significant indexes in investigating the depression-like behaviors and the pathogenesis of depression. The findings for association of BMI in key white matter (WM) tracts that are crucial to mood regulation and neurocognitive functioning has suggested that BMI might contribute to the pathophysiology of bipolar disorder (BD) through a detrimental action on structural connectivity in critical cortico-limbic networks (41). The precise mechanism and depressive-like behaviors of depression remain to be further verified by detailed evidence in our study.

Referring to investigations of recent studies, SPT was employed to evaluate the condition of anhedonic-like behaviors of rats. Anhedonia was expressed by reduced sucrose preference. In this study, significant difference was also found between fluoxetine and acupuncture groups at 7 days of the experimental procedure, which might be due to the difference in onset time between fluoxetine and acupuncture. Interestingly, significant differences was found in ameliorating ahedonia (the core symptom of depression) and mood between fluoxetine and acupuncture, suggesting the comprehensively antidepressant response of acupuncture (10, 42). Importantly, our findings indicated that fluoxetine reduced the sucrose preference at 7 days when compared with acupuncture, which might be due to the delaying onset time and side effect of fluoxetine.

The OFT was performed to evaluate the ability to adapt to new environments of the rats. The horizontal motion score and vertical motion score were scored. The CUMS model was first successfully established by Willner et al. in 1957 to simulate the exogenous factors for the onset of depression, including reduced sensitivity to reward, lack of pleasure, and behavioral and spiritual malaise. The OFT can be used to verify the degree of horizontal activity of the rat, a reduction in which is a clinical sign associated with human depression. In this study, observation of the locomotor activity scores showed that the self-regulation and the locomotor activity scores of the rats were markedly reduced integrally at 7, 14, 21, and 28 days. However, significance was found among the four groups at different time points in spite of descending tendency. The integral tendency is consistent with the reported studies. Of note, the result of the tendency in this study is in contrast to studies that showed a stress-induced decrease in locomotor activity scores. And locomotor activity score in control group decreased over time as well. There is no obvious reason for the decreased locomotor activity scores following experimental procedures, but it has been suggested to be the result of adaptation to chronic stress and changes of the external circumstance. This paradoxical effect of the descending tendency on locomotor activity scores in this study could possibly be ascribed to the different stress protocols, since different durations and intensity of adaptation have different effects on locomotor activity score. Further studies are performed to verify this tendency.

As a fundamental part of traditional Chinese medicine, acupuncture therapy has been widely considered to be an effective alternative therapy in the clinical practice. Data from our previous clinical practice and experimental investigation have investigated the antidepressant effect of acupuncture (10, 11), which is in consistent with studies that identified the safety and effectiveness of acupuncture therapy in treating MDD and post-stroke depression. Presently, the acupoint module of *Baihui* (GV 20) and *Yintang* (GV 29) was selected as the acupuncture intervention to explore the epigenetic mechanisms underlying the antidepressant effect *via* regulating the methylation level of the BDNF promoter I and BDNF expression. The findings indicated that acupuncture at *Baihui* (GV 20) and *Yintang* (GV 29) exerted significant antidepressant effect. This consistent effect of acupuncture on stress-induced depression in this study could possibly be ascribed to the function of the acupoint module since *Baihui* (GV 20) and *Yintang* (GV 29) are affiliated to the governor meridian and associated with the brain through channels and collaterals. Notably, our previous studies have provided evidence that acupuncture at *Baihui* (GV 20) and *Yintang* (GV 29) could well alleviate depression by increasing the expression of excitatory neurotransmitter in the hippocampus, attenuating impaired neurogenesis, and inhibiting the apoptosis of hippocampal neurons (10, 11). Accordingly, acupuncture therapy could be considered an alternative option for the treatment of depression. The precise mechanism underlying the antidepressant effect remains to be further determined.

### DNA Methylation and Histone Modification of BDNF in Depression

Subsequent findings from this study identified that the stress-induced decrease in serum and hippocampal BDNF protein and BDNF mRNA were in parallel, indicating its important role in the pathogenesis of depression and the synchronous expression level of central and peripheral BDNF alterations (Figure 4G). Of note, we have also identified that acupuncture exerted antidepressant effects and positively modulated the expression level of BDNF. Through analyzing the expression levels of BDNF mRNA, acH3K9 and HDAC2, and the DNA methylation level of BDNF promoter I at relative CpG sites in hippocampus, we identified that DNA methylation and histone modifications of BDNF in the hippocampus were involved in the pathological process of depression (Figures 4H,I). The CUMS procedures elevated the expression level of HDAC2 and induced the downregulation of acH3K9 protein and BDNF mRNA, and ultimately contributed to depressive disorder. Importantly, we also identified that acupuncture could well alleviate depressive-like behaviors and regulate the DNA methylation and histone modifications of BDNF in the hippocampus. Such an antidepressant effect of acupuncture was evidenced by downregulating the expression level of HDAC2, thereby promoting the expression level of acH3K9, and elevating the expression levels of BNNF mRNA and protein, and finally restoring mood.

In this study, changes in serum BDNF levels, hippocampal BDNF protein, and its mRNA levels were investigated. In the decades that have passed, since the correlation between BDNF levels and the development of depression were identified, there has been considerable progress in understanding the roles of BDNF and its contributions to the pathogenesis of depression (19, 21, 43, 44). Our study showed that serum BDNF levels, hippocampal BDNF protein, and its mRNA levels in the model group were significantly decreased following the CUMS procedures when compared with that in the control group. These findings support previous studies suggesting that the stress-induced decrease in serum and hippocampal BDNF protein and BDNF mRNA were in parallel, and the synchronous expression level of central and peripheral BDNF alterations were evidenced (Figure 4G). Interestingly, the attenuated changes in BDNF expression following the induction of CUMS were reversed by acupuncture, indicating that acupuncture exerted antidepressant effects, and positively modulated the expression level of BDNF. Such an antidepressant effect of acupuncture was evidenced by positively modulating the expression levels of BDNF and its mRNA ranging from central to peripheral level, thereby alleviating the depressive-like behaviors.

Epigenetic mechanisms, including DNA methylation, histone modifications, and non-coding RNAs, are considered to be very important for BDNF expression regulation (45, 46) and involved in the pathogenesis of depression (23, 24, 28, 47). Data from previous studies have provided compelling evidence that BDNF promoters’ methylation level and histone modifications are associated with the development of depression (28, 48–50). In this study, the expression levels of BDNF mRNA, acH3K9 and HDAC2, and the DNA methylation level of BDNF promoter I at relative CpG sites (including CpG2, CpG4, CpG 5, CpG6, CpG10, CpG11, CpG12, CpG13, CpG14, CpG15, CpG16, CpG18, and CpG24) in the hippocampus were investigated. We found that the CUMS procedures elevated the expression level of HDAC2 and induced the downregulation of acH3K9 protein and BDNF mRNA, which might contribute to the development of depression. This finding indicates that there might be somewhat consistent with a previous study, suggesting that histone modifications are of great significance for the BDNF expression regulation (45, 47). Notably, we have also investigated that acupuncture could attenuate the expression level of HDAC2, then elevate the expression level of acH3K9, and the expression levels of BNNF mRNA and protein, and ultimately restore mood (Figures 4H,I). Preliminary results from our study demonstrate that the antidepressant response of acupuncture might be mediated by regulating the DNA methylation and histone modifications of BDNF in the hippocampus. Dynamic alterations in DNA methylation and recruitment of histone deacytelases are considered to be important mechanisms in contributing to gene expression. Recent evidence has identified the methylation level of relative CpG islands associated with promoter I of BDNF in depression, in which the changes of DNA methylation of BDNF were postulated as a biomarker of major depression (28, 50).

### The Role of DNA Methylation of BDNF in Depression

Epigenetic alterations as DNA methylation, histone modifications, and non-coding RNAs are considered to be strongly associated with pathogenesis of depression with a large number of studies having examined the association between the BDNF methylation level and certain psychological diagnoses. Epigenetic mechanisms have been shown to be very important for BDNF expression regulation. Recently, various studies have associated the DNA methylation level of BDNF promoters with development of various neuropsychiatric disorders. It has been shown that there is an association of BDNF DNA methylation and reduced WM integrity in the anterior corona radiata in major depression (51).

Numerous studies have analyzed different regions within promoter I and promoter IV. It has been demonstrated that the promoter I region is differently methylated in major depression and BDs patients (52). These partly overlap with the region differently methylated in various neuropsychiatric disorders, including depression, anxiety disorder, and schizophrenia. The overlapping regions within BDNF promoter I and promoter IV may be of special interest as possible biomarker of psychiatric diseases, taking into consideration that several independent studies reported about the differences in the methylation level of these regions. Data from the previous investigation have indicated that there is a significant correlation between the ventral prefrontal cortex and quadriceps for the methylation levels of BDNF promoter I (53). Similarly, the BDNF promoters’ DNA methylation changes of BDNF promoter I and promoter IV in brain has been demonstrated to be associated with major depression and suicide. Collectively, no definite correlation between the BDNF promoters’ methylation and BDNF protein levels, although numerous methylation level differences was found, respectively in BDNF promoter I and promoter IV between various neuropsychiatric disorders (54).

During our previous studies, we investigated the epigenetic mechanisms underlying the antidepressant effect of acupuncture by regulating the methylation level of the analyzed region inside of BDNF promoter I and BDNF expression in depth. However, no significant differences in the DNA methylation level of BDNF promoter I at relative CpG sites have been found among control, model, acupuncture, and fluoxetine groups recently. This paradoxical association of the BDNF protein level with the DNA methylation level in this study could possibly be ascribed to being conditioned by other regulators of BDNF expression: particularly histone modifications, miRNAs, antiBDNF transcripts, formation of dsRNA duplexes with BDNF transcripts, alternative splicing, and posttranscriptional cleavage. Importantly, our study have provided the first evidence that the changes in histone modifications levels of acH3K9 and HDAC2 affected the BDNF expression changes both on transcript and protein levels. Accordingly, we postulate that whether the delicate alterations of the DNA methylation level of BDNF might trigger the expressions levels of BDNF both on transcript and protein levels.

## Conclusion

Considering the present findings, the epigenetic mechanisms in the antidepressant effect of acupuncture have been associated with the potential of DNA methylation and histone modifications of BDNF. However, our preliminary findings should be viewed in light of several limitations. No acupuncture control was present, thus limiting the generalizability and the interpretation of the findings in the antidepressant effect. Furthermore, only the DNA methylation level of BDNF promoter I was intensively investigated, although no significant differences in the DNA methylation level of BDNF promoter I at relative CpG sites. This paradoxical association of the BDNF protein level with the DNA methylation level in this study could possibly be ascribed to being conditioned by other regulators of BDNF expression: particularly histone modifications, miRNAs, antiBDNF transcripts, formation of dsRNA duplexes with BDNF transcripts, alternative splicing, and posttranscriptional cleavage. Further research is needed to clarify the precise mechanisms whether the DNA methylation level of BDNF promoter I, promoter II, and promoter IV is involved in the pathogenesis of depression by applying bisulfite sequencing in our future study. As highlighted above, the epigenetic mechanisms in the antidepressant effect of acupuncture have been associated with the potential of DNA methylation and histone modifications of BDNF, which could monitor the severity of symptoms and antidepressant effect of acupuncture. Strengths of this study might shed new light on conceptual frameworks of prospects for new therapies in the treatment of depression.

## Ethics Statement

All experimental procedures were in full observance of the International Association for the Use of Animals in Research and approved by the Institute of Animal Care Committee of the Beijing University of Chinese Medicine (Permit No. Kj-dw-32-20151012).

## Author Contributions

TB designed research. XZ, HJ, JL, HM, XY, BZ, and YS performed research. XZ and HJ analyzed data. HJ wrote the paper.

## Conflict of Interest Statement

The authors declare that the research was conducted in the absence of any commercial or financial relationships that could be construed as a potential conflict of interest.
